# Early-Age Hydration Reaction and Strength Formation Mechanism of Solid Waste Silica Fume Modified Concrete

**DOI:** 10.3390/molecules26185663

**Published:** 2021-09-17

**Authors:** Tao Luo, Cheng Hua, Qiang Sun, Liyun Tang, Yu Yi, Xiaofeng Pan

**Affiliations:** 1 Shaanxi Key Laboratory of Safety and Durability of Concrete Structures, Xijing University, Xi’an 710123, China; huacheng19970609@163.com (C.H.); yiyu1105@163.com (Y.Y.); panxiaofeng202104@163.com (X.P.); 2 College of Geology and Environment, Xi’an University of Science and Technology, Xi’an 710054, China; 3 Shaanxi Provincial Key Laboratory of Geological Support for Coal Green Exploitation, Xi’an 710054, China; 4 School of Architecture & Civil Engineering, Xi’an University of Science and Technology, Xi’an 710054, China; tangly@xust.edu.cn

**Keywords:** solid waste silica fume, early-age, LF-NMR, hydration reaction, compressive strength

## Abstract

Solid waste silica fume was used to replace fly ash by different ratios to study the early-age hydration reaction and strength formation mechanism of concrete. The change pattern of moisture content in different phases and micro morphological characteristics of concrete at early age were analyzed by low field nuclear magnetic resonance (LF-NMR) and scanning electron microscope (SEM). The results showed that the compressive strength of concrete was enhanced optimally when the replacement ratio of solid waste silica fume was 50%. The results of LF-NMR analysis showed that the water content of modified concrete increased with the increase of solid waste silica fume content. The compressive strength of concrete grew faster within the curing age of 7 d, which means the hydration process of concrete was also faster. The micro morphological characteristics obtained by SEM revealed that the concrete was densest internally when 50% fly ash was replaced by the solid waste silica fume, which was better than the other contents.

## 1. Introduction

Silica fume is an ultrafine powder produced during the smelting of ferrosilicon alloy or industrial silicon, and its main component is amorphous silicon dioxide. In 2018, China produced 2 million tons of silica fume, and its utilization rate was less than 60% in that year [1,2,3]. Large-scale production ferrosilicon alloy and industrial silicon production, and the resultant direct discharge of the generated dust silica fume into the atmosphere or its direct storage, harms the environment and eventually human health. Therefore, increasing the rate of recycling and reuse of silica fume byproduct is of great importance.

Since the 1990s, silica fume has been utilized in cement, concrete, refractory materials, chemicals, environmental protection and other fields. By studying the role of MgO in the thermal behavior of MgO-silica fume pastes, Li et al. [4] established that the microstructure of the MgO-silica fume mixed slurry calcined at 1450 °C is denser than other fume pastes and suitable for a new generation of refractory castables. Goodarzi et al. [5] studied silica fume as an industrial waste to assess the geomechanical and microstructural performance of modified expansive clayey soil. The results show that silica fume can improve the efficiency of soil stability.

As a highly effective pozzolanic material, silica fume is the most widely used pozzolan in the field of concrete making. As early as 1982, Norway used silica fume concrete for the construction of the Vonoves Dam [6]. Adding 10–15% silica fume to replace cement in concrete can improve the hardening performance in terms of early compressive strength, tensile strength, bending strength and elastic modulus, and it also results in concrete with higher toughness and bonding strength [7,8,9,10]. Memon et al. [11] studied the influence of silica fume on the fresh and hardened properties of fly ash-based self-compacting geopolymer concrete, and showed that when fly ash is replaced by silica fume, the workability of the concrete mixture becomes reduced, and the mechanical properties of the hardened concrete significantly improve with the amount of silica fume. Bhalla et al. [12] employed the ultrasonic pulse velocity (UPV) and ultrasonic guided waves (UGW) testing based on wave propagation to monitor the early hydration of silica fume concrete. The content of silica fume was increased from 6% to 12%, and the compressive strength of all ages was subsequently improved along with the development of dense microstructure and increase in hydration products. Negi et al. [13] studied the effectiveness of electro-mechanical impedance (EMI) technology to study embedded zirconium titanate (PZT) patches in different directions to monitor concrete hydration. However, due to a large attenuation of the ultrasonic body wave propagation in concrete, the embedded PZT patch monitoring of the concrete causes the internal damage of concrete; therefore, this technique has limitations in detecting the hydration reaction at different curing ages. Kachanov et al. found that the dielectric properties are related to the pore structure and moisture content characteristics, and they reflect the formation of the microstructure and the hydration reaction process [14,15,16]. For early age concrete, however, there is very limited information on monitoring the hydration process based on the measurement of dielectric properties by the GPR method [17,18,19,20].

The physical properties and chemical composition of silica fume are similar to that of fly ash, while silica fume has higher pozzolanic activity than fly ash [21]. The active ingredient SiO_2_ content of the solid waste silica fume used in this paper is 86.3%, while the active ingredient SiO_2_ content of common silica fume in the market is usually more than 90%. In terms of financial, the solid waste silica fume used in this paper has a lower cost. Predecessors have used a variety of methods to study the process of concrete hydration. However, those studies mainly focused on the hydration products, hydration exotherm, etc. [22,23,24], and there is very little research available on water changes during the hardening process of concrete. During the early age of concrete hydration, the distribution characteristics and transformation laws of water have a crucial impact on the hydration reaction rate in concrete, and can be used to explain the formation mechanism of compressive strength. In addition, the use of low field nuclear magnetic resonance technology (LF-NMR) to measure the changing law of concrete water content has the advantages of small sample size and non-destructiveness [25]. In this study, silica fume comes from industrial solid waste was added to the fly ash-cement composite cementitious material to study the early-age hydration reaction and strength formation mechanism of concrete. The changing law of water content during hardening of concrete was analyzed by using LF-NMR. This research aims at adding industrial solid waste silica fume into concrete by replacing part of fly ash, hence it is of great environmental protection significance and has many economic benefits. In addition, the early-age strength of concrete is a controlling factor for the safety of concrete structure construction; therefore, it also plays an essential role in actual concrete engineering applications.

## 2. Experimental Program

### 2.1. Raw Materials and Mix Proportions of Concrete

In this study, the cement used was Conch brand P·O 42.5 ordinary Portland cement produced by Liquan Conch Cement Plant in Xianyang City, Shaanxi Province, China, with its physical and mechanical properties as shown in Table 1.

**Figure 1:**
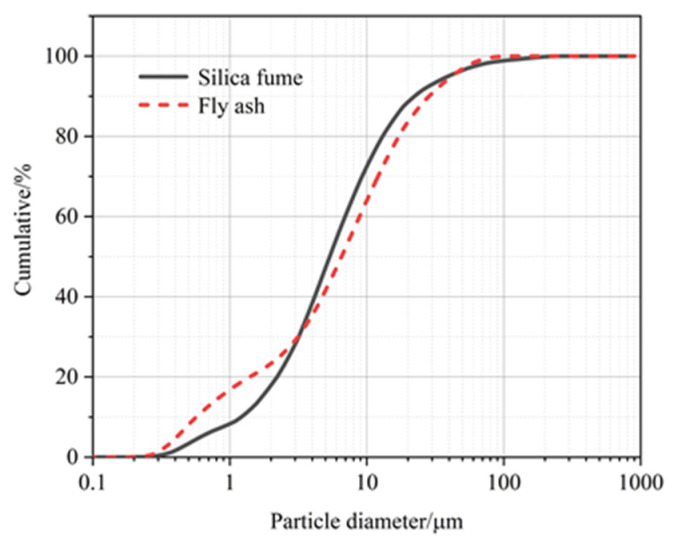
Particle size distribution of silica fume and fly ash.

The silica fume originated from the northern part of Shaanxi Province. The Grade I fly ash used was produced by Shaanxi Weihe Power Plant. Using the Bettersize 2600 laser particle size analyzer, the volume-based average particle size of silica fume and fly ash were measured as 12.35 μm and 12.71 μm, respectively. The particle size distribution is presented in Figure 1, and the physical properties of fly ash and silica fume are shown in Table 2, and the main chemical components of cement, fly ash and silica fume are shown in Table 3.

Natural river sand with a fineness modulus of 2.66 was used as fine aggregate, with an apparent density of 2630 kg/m^3^ and a bulk density of 1480 kg/m^3^. Limestone with continuous grading between 5 mm and 20 mm was used as coarse aggregate, with an apparent density of 2835 kg/m^3^ and a bulk density of 1720 kg/m^3^.

The superplasticizer applied was the Q8086 powder high performance water reducing agent produced by Shaanxi Qinfen Building Materials Co., Ltd., (Weinan, China) with a water reducing rate of 33%, air content of 2.0%, and solid content of 40%. The water reducer comprised 0.5% of the weight of the cementitious material.

The water used was ordinary tap water from Xi’an, Shaanxi Province, China.

In the experiment, five different composite cementitious material system concretes were prepared, while the external admixture method was used to keep the amount of cement unchanged. The control group without silica fume was designated as group A. In the other four groups, 25%, 50%, 75%, and 100% of fly ash were replaced with silica fume, which were numbered B, C, D, and E, respectively. The water–binder ratio was 0.48, and the mix proportions of concrete were as shown in Table 4.

### 2.2. Sample Preparation and Experimental Methods

**Figure 2:**
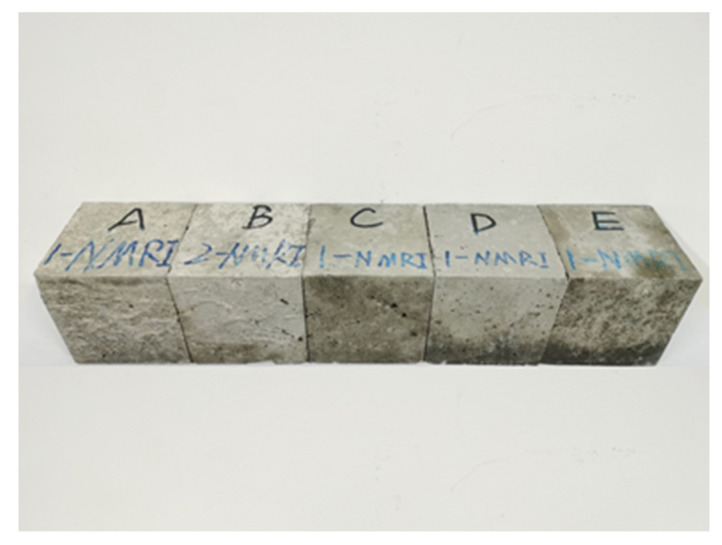
LF-NMR test blocks.

**Figure 3:**
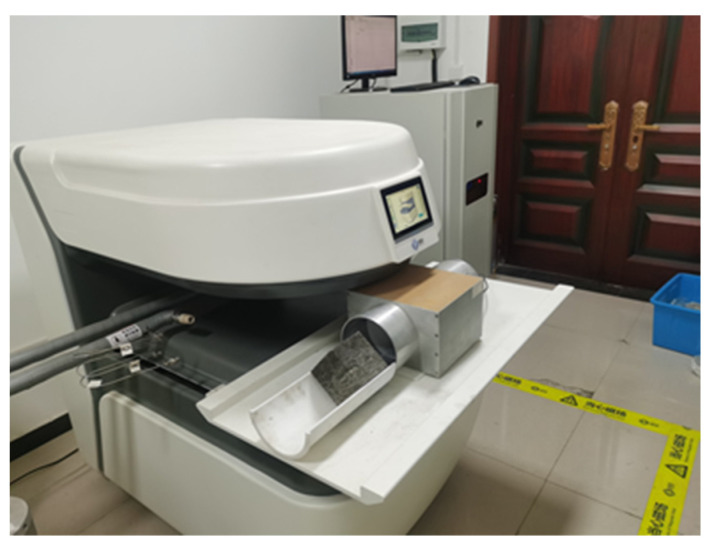
LF-NMR instrument.

(1) Prior to preparing the concrete specimens, the raw materials were weighed according to the pre-determined mixing ratios and placed in the test pan. At the same time, the superplasticizer was poured into the water to fully dissolve, the twin-shaft concrete mixer was cleaned, and the mortar with the same water-binder ratio was prepared. The remaining slurry was poured into the wall-hanging slurry in the mixer. Next, coarse aggregate, cement, silica fume, fly ash, and fine aggregate were added in sequence and stirred for 60 s. Finally, the water mixed with superplasticizer was evenly poured into the mixer and stirred for 120 s.

(2) After unloading, the slump of fresh concrete was measured according to the Chinese standard “Standard for Test Methods for the Performance of Ordinary Concrete Mixtures” (GB/T50080-2016), and the HC-7L direct-reading concrete air content tester was employed to measure the air content of fresh concrete.

(3) The remaining concrete mixture was put into a 100 mm × 100 mm × 100 mm cube mold, placed on a vibrating table for vibrating compaction, and vibrated until the surface continued to produce slurry. Each group of A, B, C, D, E made 28 specimens, totaling 140 specimens.

(4) The concrete specimens were demolded 24 h after production, and 25 specimens of each mixing ratio group were naturally cured at a curing temperature of 20 ± 2 °C and relative humidity of 95%. The WAW-1000KN universal testing machine was used to test the compressive strength of concrete cubes with different amounts of substituted silica fume at curing ages of 1 d, 3 d, 7 d, 14 d, and 28 d.

(5) The remaining three specimens in each group of mix proportions were wrapped with a polyethylene film to prevent the entry and evaporation of water, as shown in Figure 2. LF-NMR microstructure analysis and imaging was performed by the model MacroMR12-150H-I produced by Suzhou Niumag Analytical Instrument Corporation, as shown in Figure 3. LF-NMR aims to analyze the composition of a test sample based on the nuclear magnetic resonance characteristics of the hydrogen nucleus, low field nuclear magnetic resonance spectroscopy was carried out on the concrete specimens at the curing ages of 1 d, 3 d, 7 d, 14 d, and 28 d, the CPMG pulse sequence data were collected, and perform inversion calculations through *T_2_* inversion software. Thus, a spectrum composed of the LF-NMR signal amplitude and relaxation time of different types of water in the concrete test block is obtained [26].

**Figure 4:**
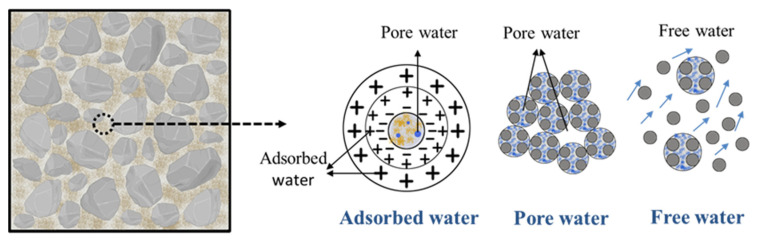
Classification of water forms in concrete.

In the hardening process of concrete, cement hydration consumes part of the physically bound water, thereby generating chemically bound water. Jehng measured the apparent transverse relaxation time of the chemically bound water in cement paste to be 12 μs [27]. It is generally assumed that physically bound water in concrete mainly includes three forms: adsorbed water, pore water and free water [28], as shown in Figure 4.

The *T_2_* spectrum includes three relaxation peaks, which are defined as peak 1, peak 2, and peak 3 from left to right, which correspond to the LF-NMR signals of hydrogen nuclei in adsorbed water, pore water, and free water, respectively [29]. The change in the peak area of the *T_2_* spectrum reflects the change in the contents of adsorbed water, pore water and free water in the filler slurry.

According to the volume and signal of the sample, a unit volume nuclear magnetic signal can be obtained, as shown in Equation (1). Then, the total water content of the tested sample can be obtained according to the calibration formula in Equation (2) [30].

(1) $$y=\frac{A}{V}$$

(2) y=299.806x+54.227

where y is the unit volume nuclear magnetic signal, *x* is the total water content, *V* and *A* are the volume and signal of the sample, respectively.

(6) The JSM-7610F field emission scanning electron microscope produced by JEOL was utilized to test the surface micromorphology of 28-day-old concrete. Before the test, the sample was sprayed with gold and vacuumed for inspection by the scanning electron microscope imaging system.

## 3. Results

### 3.1. Working Performance of Fresh Concrete

The working performance of fresh concrete for each mixing ratio is shown in Table 5. With the increase in the replacement amount of silica fume, the slump of fresh concrete gradually decreased, and the air content showed a trend of first decreasing and then increasing. The slump of each group of fresh concrete was not less than 10 mm, which meets the Chinese standard “Standard for Test Methods for Performance of Ordinary Concrete Mixtures” (GB/T50080-2016).

### 3.2. Compressive Strength of Early-Age Concrete Cubes

#### 3.2.1. Influence of Age on the Compressive Strength of Concrete Cubes

**Figure 5:**
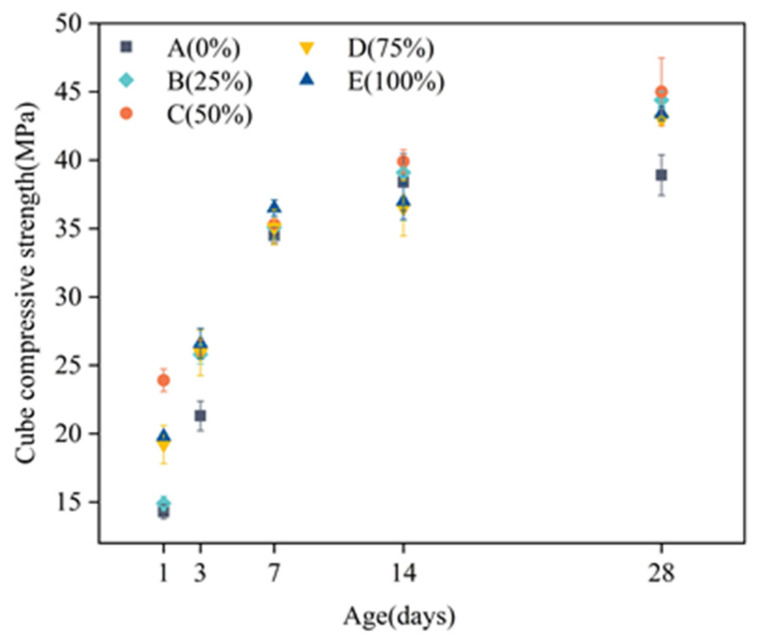
Relationship diagram of the compressive strength of concrete cubes at different ages.

Figure 5 presents the relationship between the compressive strength of concrete cubes at different ages. It can be seen from Figure 5 that, over the course of time, the compressive strength of concrete cubes of each mixing ratio group showed a nonlinear growth trend. Before 7 days of age, the compressive strength of each mixing ratio group increased rapidly. Within 7 to 14 days of age, the compressive strength growth trend of A, B, and C groups slowed down slightly, and the compressive strength growth rate of D and E groups tended to stabilize. After 14 days of age, the growth rate of compressive strength of group A slowed down and tended to converge, the growth trend of compressive strength of groups B and C slows down slightly, and the growth rate of compressive strength of groups D and E rebounded.

#### 3.2.2. Effect of the Replacement Amount of Silica Fume on the Compressive Strength of Concrete Cubes

**Figure 6:**
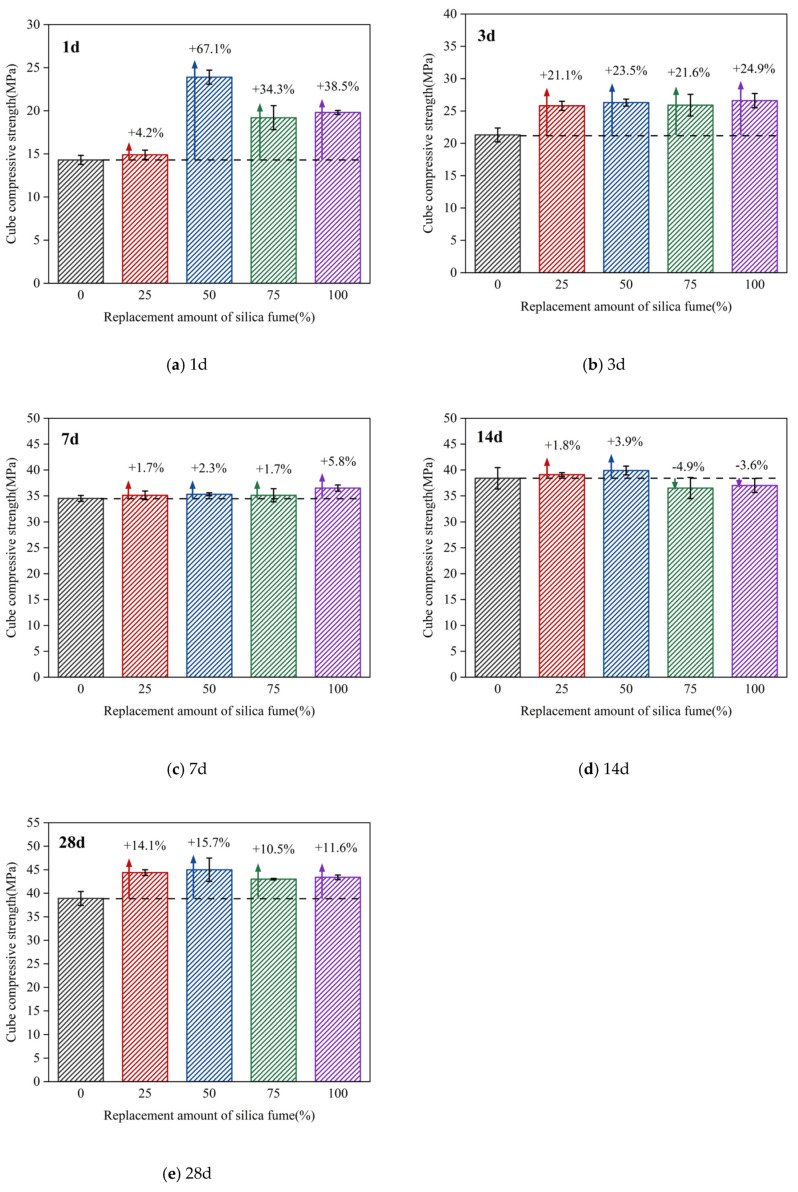
Comparison of compressive strength of concrete cubes with different replacement amounts of silica fume at different curing ages: (**a**) 1 d; (**b**) 3 d; (**c**) 7 d; (**d**) 14 d; (**e**) 28 d.

Figure 6 is a comparison diagram of the compressive strength of concrete cubes of different ages with different amounts of silica fume replacement. Figure 6 shows that, at the age of 1 d, compared with the standard mix proportion without silica fume addition, the cubic compressive strengths of concrete in groups B, C, D, and E increased by 4.2%, 67.1%, 34.3%, and 38.5%, respectively. At the age of 3 d, the cubic compressive strengths of concrete in groups B, C, D, and E increased by 21.1%, 23.5%, 21.6%, and 24.9%, respectively. At the age of 7 d, the cubic compressive strengths of concrete in groups B, C, D, and E increased by 1.7%, 2.3%, 1.7%, and 5.8%, respectively. At the age of 14 d, the cubic compressive strengths of concrete in groups B, C, D, and E increased by 1.8%, 3.9%, −4.9%, and −3.6%, respectively. At the age of 28 d, the cubic compressive strengths of concrete in groups B, C, D, and E increased by 14.1%, 15.7%, 10.5%, and 11.6%, respectively. With the increase of silica fume replacement amount, the cubic compressive strength of concrete showed a trend of first increasing, then decreasing and increasing again. For the age of 3 d, the cubic compressive strength of concrete with different silica fume substitution amounts was greatly improved, and the growth rate of the compressive strength of concrete with a silica fume substitution of 50% (group C) increased by 67.1%. For the ages of 7 d and 14 d, the compressive strength of concrete was slightly increased, and the increase amplitude was within 5%. For the age of 28 d, the compressive strength of concrete continued to increase by more than 10%.

### 3.3. Hydration Reaction of Early-Age Concrete

#### 3.3.1. Effect of Silica Fume Replacement Amount on the Hydration Reaction

**Figure 7:**
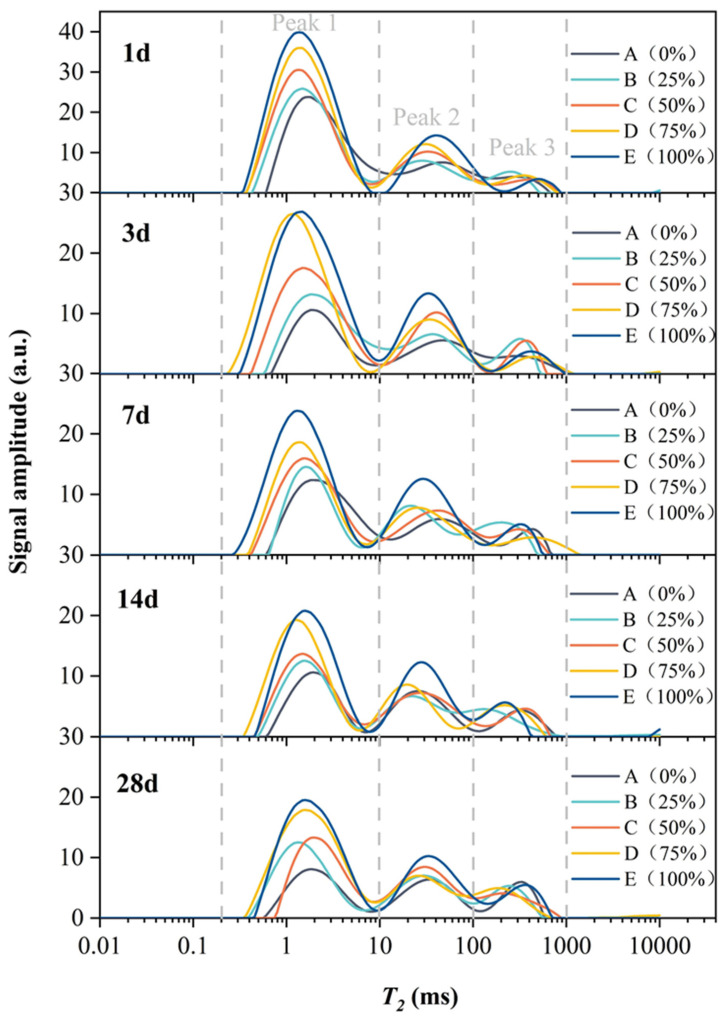
Evolution of *T_2_* spectra of concrete fluids with different silica fume replacement amounts at different ages.

Figure 7 shows the evolution of *T_2_* spectra of concrete fluids with different amounts of silica fume substitution at different ages. As seen in the figure, the total water content in the concrete shows a gradual rising trend with the increase of the replacement amount of silica fume. The area of peak 1 shows an increasing trend with more water is adsorbed, indicating that water in the concrete mainly exists in the adsorbed form. The area of peak 2 generally shows an increasing trend with a higher volume of pore water. The area of peak 3 presents a decreasing trend, indicating that the free water in the concrete decreases with the rise of silica fume replacement amount.

#### 3.3.2. Influence of Age on Hydration Reaction

**Figure 8:**
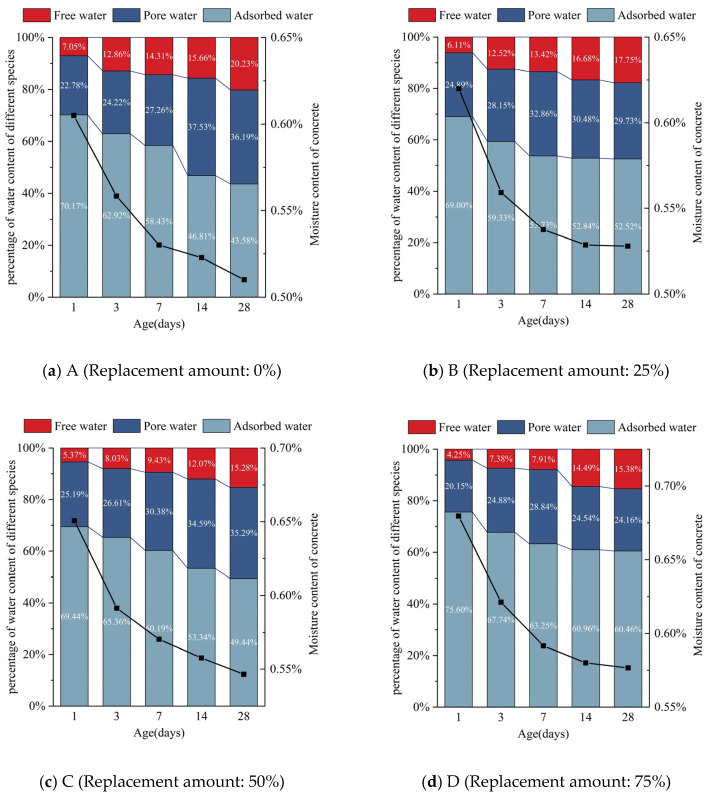

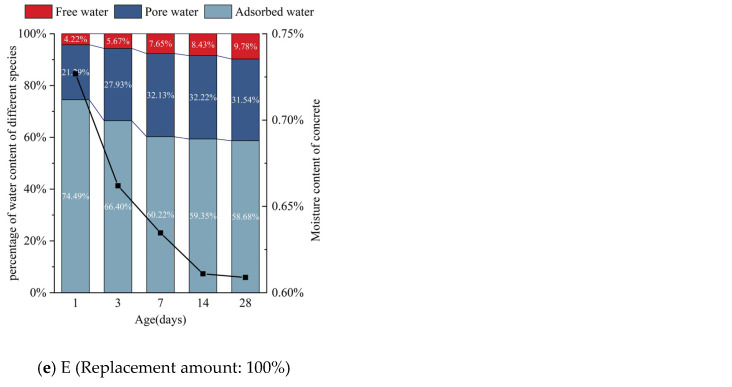
Different forms of water content and total water content in concrete of different ages: (**a**) A (Replacement amount: 0%); (**b**) B (Replacement amount: 25%); (**c**) C (Replacement amount: 50%); (**d**) D (Replacement amount: 75%); (**e**) E (Replacement amount: 100%).

The graph of different forms of water content and total water content in concrete with different amounts of silica fume replacement is presented in Figure 8. It is clearly seen that the area of peak 1 gradually decreases with hydration time. The observed decline is relatively large before reaching 7 d for each mix ratio. Subsequently, the peak area slightly decreases. This shows that the volume of adsorbed water in the concrete declines faster before 7 d; between 7 and 28 days, however, the volume of adsorption water in the concrete declines relatively slowly. The area of peak 2 fluctuates along with hydration time, but the overall change is not considerable—the relevant peak area changes little compared with the other two peaks. With the progress of hydration reaction, the area of peak 3 gradually increases. The change trend of the total peak area is similar to that of the area of peak 1. The reduction in the peak area is relatively large before 7 d, while that after 7 d is relatively small.

## 4. Discussion

### 4.1. Relationship between Changes of Cube Compressive Strength and Water Content

**Figure 9:**
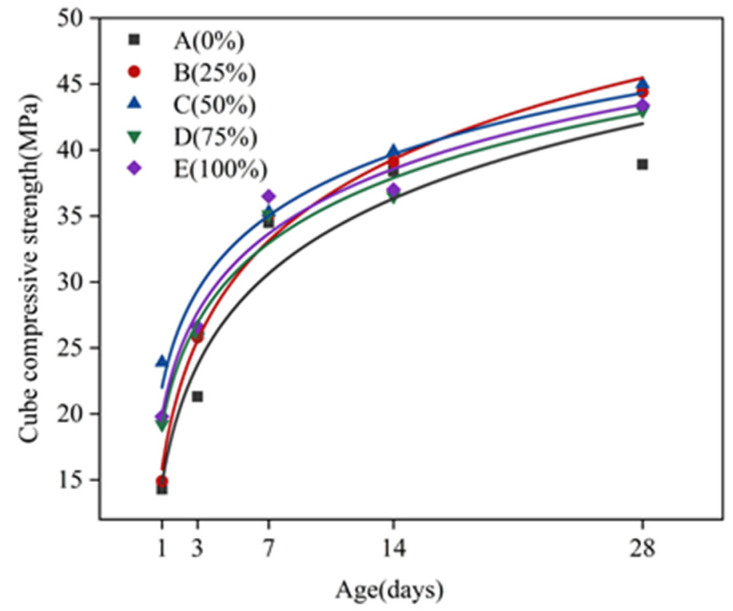
*f_cu,k_-T* fitting curve of concrete.

**Figure 10:**
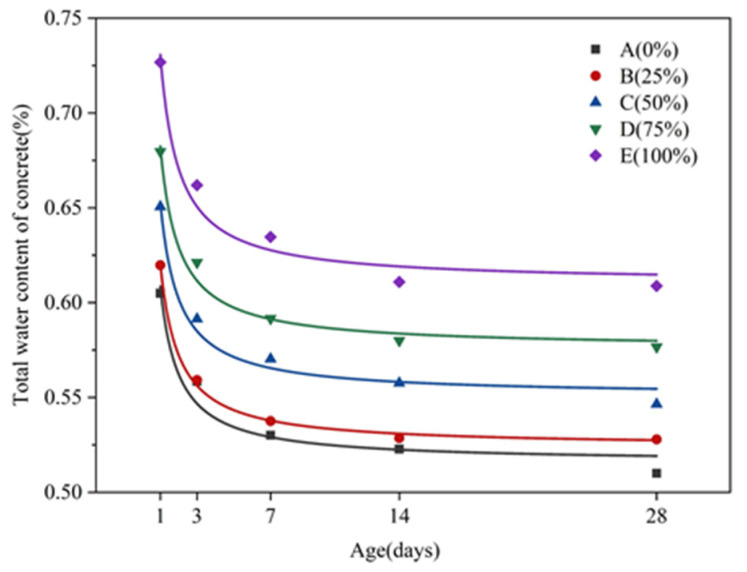
Fitting curve of total water content of concrete.

In order to better explore the relationship between the compressive strength of concrete cubes and the change of total water content over time, the least squares method was used for fitting. Figure 9 shows the fitted curve of the compressive strength of concrete cubes over time, whereas Figure 10 depicts the fitted curve of the total water content of concrete.

The correlation equations of the two parameters are as follows:

(3) $$f_{cu,k}\left(t\right)=a\mathrm{lg}\left(t\right)+b$$

(4) $$W\left(t\right)=ct^{-1}+d$$

In the relational equation, *t* represents age in days; $f_{cu,k}t$ represents the compressive strength of the concrete cube at age *t*; W(t) represents the total water content of concrete at age *t*; a, b, c, and d are constants, and their values are determined by experiment.

The coefficients of the fitting curve equation are shown in Table 6 below.

It can be seen from the fitting curve that, before 7 days of age, the compressive strength of concrete cubes in each mixture group has a trend of faster growth, while the total moisture content of concrete in each mixture group rapidly decreases. The hydration reaction is the process of converting physically bound water into chemically bound water in concrete [31]. The actual measurement of total water content is the total amount of water physically bound in concrete, indicating that the smaller the total water content, the more physically bound water will be consumed by hydration. Meanwhile, the greater the amount of chemically bound water generated, the more intense the hydration reaction, and the larger the growth trend of compressive strength of the cube will be. Within 7 to 28 days of age, the cubic compressive strength of each mixture group increases slowly. At the same time, the total water content slowly declines, indicating that the hydration reaction rate slowly weakens in the later stage, and the compressive strength of the cube gradually tends to a stable value. It can be seen that the rate of total water content reduction in the concrete can reflect the speed of the hydration reaction, which in turn can reflect the speed of the formation of compressive strength.

For concrete materials, moisture is not only a guarantee for strength development, but also closely related to performance [32]. In the early hydration process of concrete, water mainly exists in the form of adsorbed water, accounting for more than 49% of the total water content. Free water will cause bleeding on the concrete surface, and cause it to appear honeycombed and pockmarked [33]. In addition, the reduction in free water content in concrete will lead to the decreased workability of fresh concrete. When silica fume, as an ultra-fine material with a large specific surface area, is added into the concrete mixture, the free water in the concrete will be restricted by the silica fume particles, thus greatly reducing the degree of bleeding.

### 4.2. Relationship between the Formation of Cube Compressive Strength, Microstructure and Hydration Products

**Figure 11:**
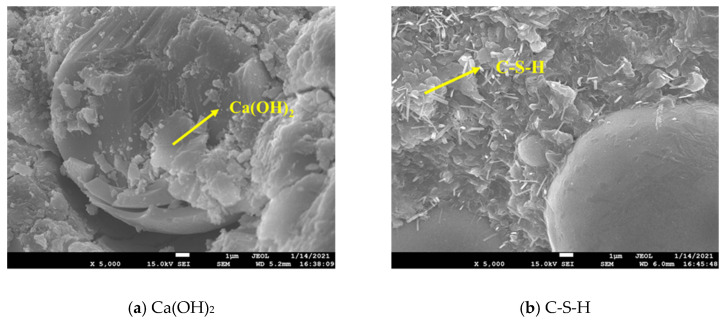
The hydration products Ca(OH)_2_ and C-S-H: (**a**) Ca(OH)_2_; (**b**) C-S-H.

The main chemical composition of silica fume is amorphous SiO_2_; high-fineness amorphous SiO_2_ has high pozzolanic activity. The hydration reaction between cement and water yields the hydration product Ca(OH)_2_, as shown in Figure 11a. SiO_2_ can quickly undergo a secondary hydration reaction with Ca(OH)_2_ under the alkaline excitation to form C-S-H gel (as shown in Figure 11b), which can fill the concrete pores, thus improving concrete strength and performance [34,35,36]. In addition, since the particle size of cement, fly ash and silica fume shows a decreasing trend, these three form a good particle gradation relationship and can fill the pores in the concrete aggregate, making the whole structure denser, which plays a significant role in improving the compressive strength of concrete [37].

**Figure 12:**
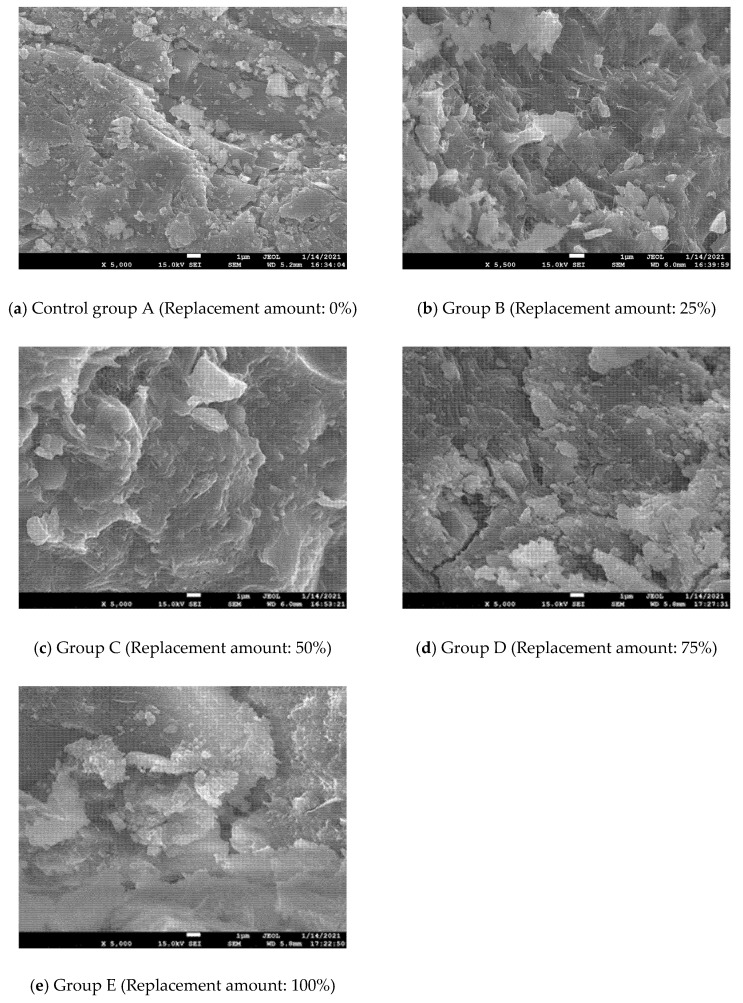
The 28th day microstructure of concrete: (**a**) Control group A (Replacement amount: 0%); (**b**) Group B (Replacement amount: 25%); (**c**) Group C (Replacement amount: 50%); (**d**) Group D (Replacement amount: 75%); (**e**) Group E (Replacement amount: 100%).

This test proves that, as a partial substitute for fly ash, silica fume can give full play to the pozzolanic effect of mineral admixtures, and is highly effective in improving the cubic compressive strength and working performance of concrete. The concrete cube has the highest compressive strength when the substitution amount of silica fume is 50% (Group C). Compared with the control group A without silicon powder, the surface of group C is covered by a more irregularly structured C-S-H gel, and the resulting microstructure also denser (as shown in Figure 12). A similar study by Dutta et al. [38] proved that mortar mixed with silica fume has better microstructure and smaller porosity. Herein, when the mass content of silica fume was 5%, the compressive strength of the silica fume-based polymer mortar could be improved.

However, in the case of a large replacement amount of silica fume (Group D, Group E), the compressive strength of concrete showed a slowed increasing trend. Although the compressive strength of such concrete cubes was higher than that of control group A cubes, it was lower than that of group C. While the D and E groups also contained C-S-H gels, the pores in the surface concrete still existed, and the C group was denser in comparison (as shown in Figure 12). Consequently, the silica fume replacement amount should not be too large, otherwise it will make the growth trend of concrete strength inconspicuous, and the strength improvement effect of silica fume addition will be diminished. In a relevant study, Memon et al. [2] found that, when the addition of silica fume exceeded 10% of the mass of fly ash, the instability of concrete specimens could lead to a decrease in concrete strength.

Suarez et al. [39] studied the role of fly ash and silica fume in the process of cement hydration, and proved that the level of C-S-H gel formation increased the fastest in the early stage and gradually dwindled over time. In our experiment, the compressive strength of concrete cubes in each mixture group increased rapidly before 7 days of age. Within 7 to 28 days of age, however, the increase of cubic compressive strength of each mixture group became slow. Therefore, the formation of compressive strength of the cube is closely related to the amount of C-S-H gel produced.

## 5. Conclusions

Silica fume-like solid waste was used for preparing composite cementitious materials based concrete by replacing 0%, 25%, 50%, 75%, and 100% of fly ash. The working performance of fresh concrete and the compressive strength of concrete cubes at different ages were tested. The contents of different types of internal water (moisture) at different ages were monitored by LF-NMR. The hydration products of concrete at age of 28 d were analyzed by using SEM. By studying the early-age hydration and strength formation mechanism of silica fume-like solid waste based composite cementitious concrete, the following main conclusions can be drawn:

(1) As the amount of solid waste silica fume increased, the slump and expansion of the concrete mixture tended to decrease and its air content firstly decreased and then increased.

(2) With the increase of age, the compressive strength of concrete at each replacement ratio of solid waste silica fume showed an increasing trend, which increased faster in the age of 7 d. Besides, the compressive strength of concrete showed an increasing-decreasing-increasing trend with the increase of the amount of solid waste silica fume at each age. The overall comparison showed that the compressive strength of concrete at all ages was enhanced best when the replacement ratio of solid waste silica fume was 50%.

(3) With the increase of the amount of solid waste silica fume, the total water content in the concrete showed a general trend of gradual increase. Additionally, with the continuation of hydration reaction, the water content of concrete under each dosage of solid waste silica fume continuously decreased, which was consistent with the change trend of adsorbed water during the curing period. Moreover, the change of the total water content and adsorbed water of concrete under each dosage was more obvious in the age of 7 d, which was shown by the phenomenon that the compressive strength of concrete grew faster in this period.

(4) Through the electron microscope analysis, it was found that the concrete was the densest and most effective in improving the internal pore structure when the content of solid waste silica fume was 50% compared to the other contents, and thus its compressive strength was the highest.

The active ingredient SiO_2_ content of solid waste silica fume used in this study is 86.3%. Most studies use high-purity silica fume. In financial terms, the silica fume used in this study has a lower cost, and the cube strength has been greatly improved. In addition, using solid waste silica fume instead of fly ash can greatly increase the early strength of concrete, which also plays an important role in the application of high early-strength concrete engineering.

## Figures and Tables

**Table 1:** Physical and mechanical properties of cement.

Cement Type	Ignition Loss (%)	Stability	Setting Time (min)		Compressive Strength (Mpa)		Flexural Strength (Mpa)	
			Initial	Final	3 d	28 d	3 d	28 d
P·O 42.5	4.00	Qualified	175	245	28.0	48.8	5.1	7.7

**Table 2:** Physical properties of fly ash and silica fume.

Material	Apparent Density (g/cm^3^)	Water Demand Ratio (%)	Moisture Content (%)	Specific Surface Area (m^2^/kg)	Ignition Loss (%)	Activity Index (%)	
						7 d	28 d
Fly ash	**2.56**	**88**	**0.40**	**994**	4.70	/	73
Silica fume	2.19	120	0.50	18,000	3.70	107	125

**Table 3:** Main chemical components of cement, fly ash and silica fume (%).

Material	SiO_2_	Al_2_O_3_	CaO	Fe_2_O_3_	MgO	SO_3_
Cement	22.81	5.62	61.43	3.36	1.35	2.17
Fly ash	49.02	31.56	4.88	6.97	0.83	1.2
Silica fume	86.30	-	-	-	-	-

**Table 4:** The mix proportions of concrete (kg/m^3^).

Code	Replacement Amount of Silica Fume	Silica Fume	Fly Ash	Cement	Coarse Aggregate			Sand	Water	Superplasticizer
					Small	Medium	Large			
A	0%	0	48	192	434	434	577	620	115	1.2
B	25%	12	36	192	434	434	577	620	115	1.2
C	50%	24	24	192	434	434	577	620	115	1.2
D	75%	36	12	192	434	434	577	620	115	1.2
E	100%	48	0	192	434	434	577	620	115	1.2

**Table 5:** Working performance of fresh concrete.

Code	Slump (mm)	Air Content (%)
A	90	1.42
B	76	0.90
C	70	0.82
D	62	0.90
E	55	1.30

**Table 6:** Relation coefficients of fitted curve.

Code	a	b	*R* ^2^	c	d	*R* ^2^
A	18.851	14.717	0.9292	0.09273	0.51588	0.9590
B	20.485	15.818	0.9891	0.09656	0.52404	0.9975
C	15.421	22.004	0.9575	0.10216	0.55093	0.9800
D	16.378	19.114	0.9784	0.10634	0.57613	0.9828
E	16.261	19.926	0.9668	0.12022	0.61053	0.9690

## Data Availability

Not applicable.

## Sample Availability

Samples of the compounds not available are available from the authors.
